# Mimic for Child Physical Abuse: Biochemical and Genetic Evidence of Hypophosphatasia without Classic Radiologic Findings

**DOI:** 10.1155/2020/3246762

**Published:** 2020-11-24

**Authors:** Kasra Zarei, John A. Bernat, Yutaka Sato, Rachel Segal, Guru Bhoojhawon

**Affiliations:** University of Iowa, Carver College of Medicine, 200 Hawkins Drive, Iowa City, IA 52242, USA

## Abstract

Infants presenting with multiple fractures without a plausible accident history need to be evaluated for child abuse or underlying predisposing conditions such as osteogenesis imperfecta and hypophosphatasia. We present a case of infantile hypophosphatasia with multiple unexplained fractures but otherwise normal radiographs in the setting of biochemical and genetic evidence of hypophosphatasia. Standard screening tests for hypophosphatasia include serum alkaline phosphatase level and genetic testing. Despite the presented case's positive biochemical and genetic testing, the case did not have any other radiologic finding suggesting infantile hypophosphatasia, such as severe bone mineralization deficits and rickets. While patients with hypophosphatasia can have increased bone fragility, this has been reported in the context of radiologic abnormalities of the skeleton. Thus, this case is potentially the first reported infantile hypophosphatasia case presenting with no findings of rickets on radiographs, raising concern that the fractures and especially the radius head dislocation might be due to physical abuse.

## 1. Introduction

Unexplained fractures in young children raise concern for child abuse. Although rare, fractures occur in numerous medical conditions. One such condition is hypophosphatasia, a genetic condition caused by mutations in the *ALPL* gene which encodes tissue-nonspecific alkaline phosphatase, leading to defective bone and teeth mineralization. Hypophosphatasia spans a multitude of clinical manifestations from before birth to early adulthood, and the diagnosis is often delayed. To our knowledge, this case is the first reported case of an infant with biochemical and genetic evidence of hypophosphatasia without classic radiological findings, which was the subject of child abuse evaluation.

## 2. Case Presentation

A previously healthy full-term 6-month-old male infant was brought to the Emergency Department (ED) with an 8-hour history of not moving his right arm and marked fussiness. He had been behaving normally until a brief period when he was left unsupervised with two toddlers as the babysitter went to the kitchen to wash a bottle. Upon arrival, he had tachycardia (169 beats/minute). The rest of his vitals were normal. He could move all his extremities except the right upper extremity. He had a visible deformity of the right arm, and his neurological exam was normal.

**Figure 1:**
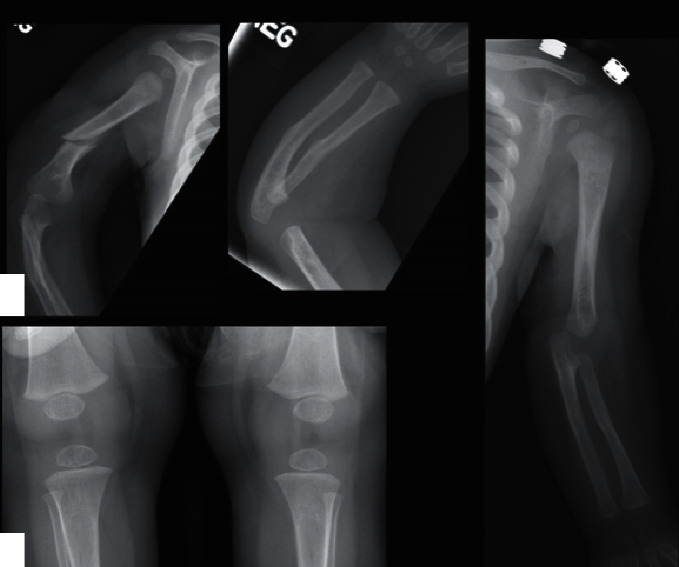
Spiral fracture of the right humerus is present (a). A right forearm radiograph shows healed midshaft fractures of the radius and ulna (b). The left upper extremity radiograph shows midshaft sclerosis and subtle cortical irregularity of the humerus, radius, and ulna, suggestive of healed fractures (c). The bone density is normal and no rarefaction of the zones of provisional calcification is present in the wrist and knees (b–d).

Radiographs of the right upper extremity revealed an acute spiral right humeral fracture and healed fracture of the right mid-radius and ulna. This prompted a comprehensive child abuse workup. A skeletal survey showed thickened cortex in the mid-shaft of left humerus, radius, and ulna with subtle cortical irregularity, suggestive of healed fractures (Figure 1). The computed tomography (CT) scan of the head was negative (not shown). The dilated fundus examination was negative for retinal hemorrhages. The patient's arm was placed in a sling, and he was admitted for further management.

Upon further history, the caretakers reported that four days prior, the patient fell out of bed onto carpeted flooring. He was initially upset but was nursed back to sleep and was at baseline after he woke up. A similar fall happened several months ago, but the patient had returned to his baseline quickly without signs of injury. Developmental history was normal. There was no family history of unexplained or infantile fractures, bone disorders, connective tissue disorders, or bleeding disorders. There was no history of domestic violence in the home. Laboratory evaluation included normal calcium, phosphorus, magnesium, copper, ceruloplasmin, parathyroid hormone, 25-hydroxy vitamin D levels, free T4, and thyroid stimulating hormone (TSH). Urine drug screen was negative for illicit substances. Genetics consultation was conducted with subsequent *COL1A1* and *COL1A2* gene testing to evaluate for osteogenesis imperfecta (OI). A next-generation sequencing panel of OI-related genes was performed by an external genetic testing laboratory. The patient was discharged to the parents on hospital day 4, per child protective services (CPS) recommendations.

OI genetic testing returned negative, but the testing laboratory reported two variants in the *ALPL* gene, a pathogenic variant (c.526G > A, p.Ala176Thr), and a likely pathogenic variant (c.119C > T, p.Ala40Val). To confirm the diagnosis of hypophosphatasia, additional testing was sent and revealed low serum alkaline phosphatase (46 U/L; normal: 122–469 U/L), high serum pyridoxal 5′-phosphate (476 mcg/L; reference range: 5–50 mcg/L), and high urine phosphoethanolamine (850 nmol/mg; reference range: 15–341 nmol/mg). Parental site-specific testing for the two *ALPL* variants revealed that the patient's mother had the p.Ala40Val variant and his father had the p.Ala176Thr variant. Based on his clinical history and laboratory findings despite the absence of classical radiographic findings, our patient was diagnosed with hypophosphatasia. On subsequent follow-up 10 weeks after discharge, he was growing well and had no new fractures. Enzyme replacement therapy was considered but ultimately not started.

## 3. Discussion

Hypophosphatasia is a rare (∼1 in 100,000) inherited disorder of bone development caused by deficiency of serum alkaline phosphatase, leading to defective bone mineralization. The clinical presentation of hypophosphatasia is variable with multiple subtypes based on timing of onset of symptoms and presentation: (1) perinatal-onset: infant is born with symptoms, (2) infantile-onset: before six months of age, (3) childhood-onset: between six months of age to less than 18 years of age, (4) adult-onset: over 18 years of age, and (5) odonto-hypophosphatasia: presents at any age with only dental manifestations [1]. Recently, a mild prenatal form has been described as a sixth subtype [2]. The severity ranges from stillbirth without mineralized bone to pathologic fractures of the lower extremities in later adulthood.

While there are many clinical, biochemical, and radiographic features suggestive of hypophosphatasia, there are no formal criteria for diagnosis [3–5]. Furthermore, clinical features seen in one subtype may overlap with other subtypes. Patients with infantile hypophosphatasia often have severe bone mineralization deficit with bowing of the limbs and small thorax and pulmonary hypoplasia or rickets [3, 4]. Childhood hypophosphatasia may present with limited mobility, chronic pain, short stature, rickets, long bone deformity, and nontraumatic fractures [6]. However, marked bone fragility during childhood is rare [2]. All types display low serum alkaline phosphatase and the presence of one or two pathogenic variants in the *ALPL* gene [7]. The presented case did have one pathogenic variant and one likely pathogenic variant in the *ALPL* gene, allowing a diagnosis giving the clinical and biochemical findings.

There is one case describing an older child with unexplained fractures and otherwise normal radiographs who was found to have biochemical evidence of hypophosphatasia [2]: this case report described a 9-year-old female with a fracture of the right tibia from jumping, unexplained fracture of the right femoral neck, and unexplained fracture of the metaphysis of the right femur [2]. There were no reported cases of infants with biochemical and/or genetic evidence of hypophosphatasia without classic radiologic findings. The patient in this case we present had biochemical and genetic evidence of hypophosphatasia and otherwise normal radiographs except for mild osteopenia with no evidence of rickets despite multiple fractures. More apparent radiographic abnormalities in hypophosphatasia include bowing deformity of the long bones, severe osteopenia, and generalized metaphyseal rickets-like changes [4]. Less apparent cases pose challenges in differentiating from nonaccidental trauma like the current case.

Bone diseases, including osteogenesis imperfecta and hypophosphatasia, can be frequently confused with child physical abuse [7, 8]. Patient history, clinical course, family history, physical examination, routine laboratory tests, and radiographic imaging all contribute to distinguishing hypophosphatasia and bone diseases from child physical abuse [8]. Multiple fractures are less typical of hypophosphatasia, but hypophosphatasia cannot be excluded from the differential diagnostic list until biochemical testing is done as illustrated in this case. Serial measurement of serum alkaline phosphatase activity is usually sufficient to identify hypophosphatasia [7].

It is also important to consider child physical abuse when hypophosphatasia is the working diagnosis, which is not always the practice [4, 5, 9]. Systematic reviews of the literature only identified a handful of studies that directly compared and contrasted child abuse with metabolic or genetic bone disease in the same study [8, 10–15]. Of the five studies identified, four of them focused on cases of osteogenesis imperfecta [11–14]. To our knowledge, only one study directly mentioned the consideration of child abuse in the differential diagnosis of hypophosphatasia [8, 10]. Infants presenting with multiple fractures without a plausible accident history need to be evaluated for child physical abuse first and foremost while considering underlying predisposing conditions such as hypophosphatasia and osteogenesis imperfecta. It should also be kept in mind that children with a medical condition may also be abused or abuse cannot be ruled out [8, 11]. The healing fractures of the left and right radius and ulna on the patient's radiographs are not typical findings in hypophosphatasia but more consistent with healing traumatic fractures rather than pathologic fractures. In addition, the bending deformities of the upper and lower extremities could be due to normal variant of neonatal period or hypophosphatasia causing a delayed resolution or due to healing inflicted fractures.

As a result of these factors, diagnostic considerations in this case may include the following: hypophosphatasia leading to decreased bone mineralization might have caused bone weakness and multiple fractures with minimal trauma. However, the literature shows that asymptomatic children with hypophosphatasia and normal-appearing bones experience fractures anywhere between the ages of 6 months and 18 years of age (anecdotally often around 8-9 years of age) [3]. All infants with the infantile form of hypophosphatasia on the other hand present with overt findings of rickets and abnormal bones that explain the bone fragility [3, 7]. Lastly, radial head dislocation observed in this child cannot be explained by hypophosphatasia alone and may be due to physical abuse. Thus, physical abuse in the context of hypophosphatasia cannot be ruled out for this child, who has at least two sets of fractures from two different timeframes involving his right arm as well as a chronic-appearing radial head dislocation of the same arm. It is also possible that the incidental diagnosis of hypophosphatasia is a mild form of the disease with little or no impact on bone fragility. Unfortunately, there is no gold standard test for diagnosing child abuse, as the diagnosis is often determined by some combination of the patient's history, physical exam findings, radiographic findings, and an investigation by a state agency.

Thus, this case is the first report of infantile hypophosphatasia presenting with no findings of rickets on radiographs, raising concern that his fractures and especially radius head dislocation might be due to physical abuse.

## Data Availability

No additional data were used to support this study.
